# Supplementary material for: The ESCRT protein CHMP5 promotes T cell leukemia by enabling BRD4-p300-dependent transcription
Source: Nat Commun. 2025 May 3;16:4133. doi: 10.1038/s41467-025-59504-9 (PMC12049546; doi:10.1038/s41467-025-59504-9)
Supplement: Supplementary file 1 — Supplementary Information [file 41467_2025_59504_MOESM1_ESM.pdf]

The ESCRT protein CHMP5 promotes T cell leukemia by enabling BRD4-p300-dependent transcription

Katharine Umphred-Wilson[1,2], Shashikala Ratnayake[3,#], Qianzi Tang[4,#], Rui Wang[4,#], Sneha Ghosh Chaudhary[1], Ballachanda N. Devaiah[1], Josephine Trichka[1,2], Jan Wisniewski[1], Lan Zhou[5], Qingrong Chen[3], Daoud Meerzaman[3], Dinah S Singer[1], and Stanley Adoro[1,*]

Affiliations: [1]Experimental Immunology Branch, National Cancer Institute, National Institutes of Health, Bethesda, MD 20892, USA; [2]Immunology Training Program, Department of Pathology, Case Western Reserve University School of Medicine, Cleveland, OH 44106, USA; [3]Computational Genomics and Bioinformatics Branch, Center for Biomedical Informatics & Information Technology, National Cancer Institute, National Institutes of Health, Bethesda, MD 20850, USA; [4]College of Animal Science and Technology, Sichuan Agricultural University; Chengdu 611130, China; [5]Department of Pathology and Genomic Medicine, Houston Methodist Hospital, Houston, TX 77030, USA. [#]These authors contributed equally.

***Correspondence:**
Dr. Stanley Adoro
Tel: +1 240-858-3217
Fax: +1 240-541-4564
Email: stanley.adoro@nih.gov

Supplementary Information

Supplementary Figures 1-8

Supplementary Tables 1-4

Supplementary References

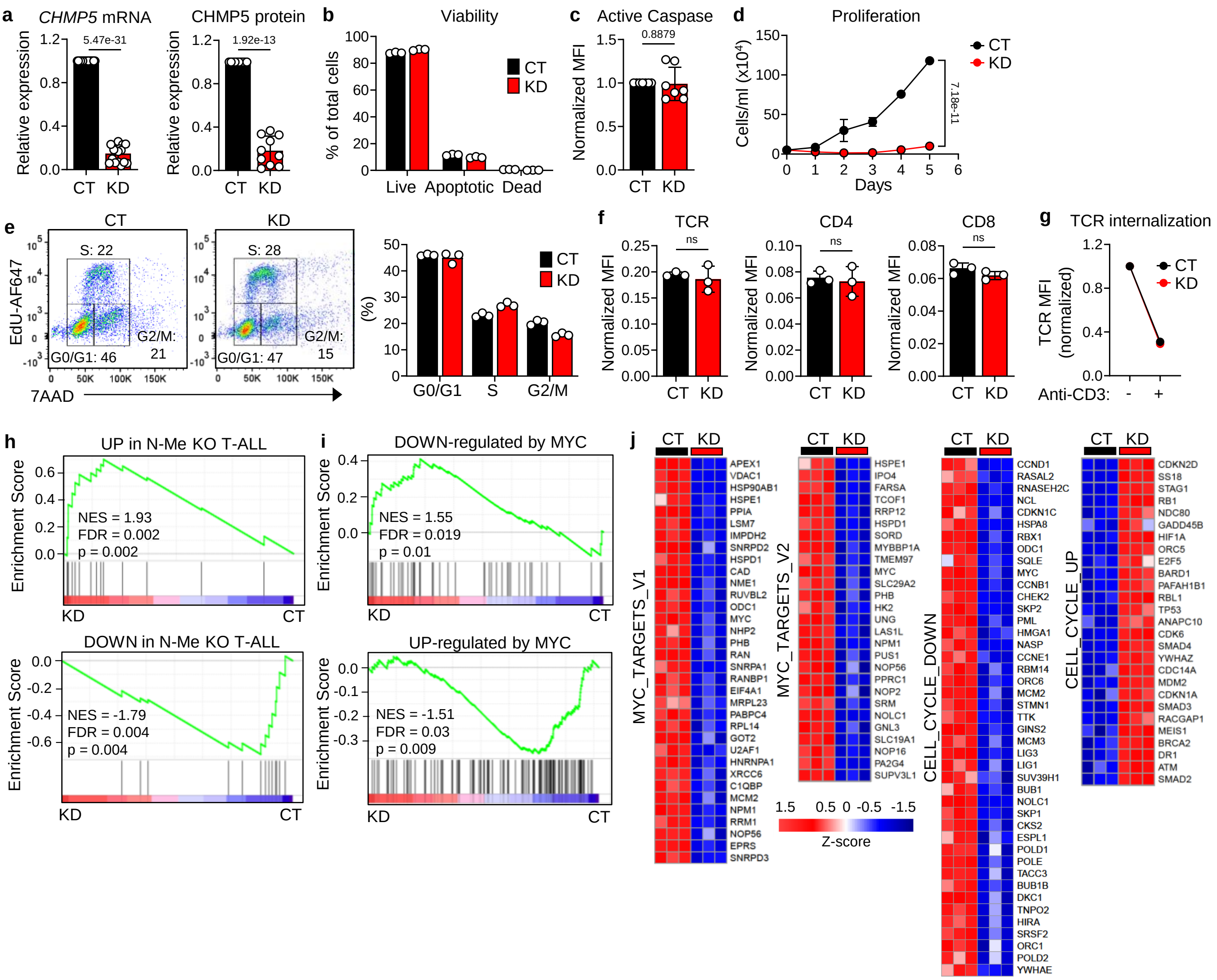

Supplementary Figure 1. CHMP5 promotes the T-ALL gene program.
a, CHMP5 mRNA and protein expression relative to Actin and normalized to CT CUTLL1 cells. Data are presented as mean ± SD of biological replicates pooled from 5 independent experiments. CHMP5 mRNA, n= 17, CHMP5 protein, n= 10. Student's t-test, two-tailed. **b,** Viability of CT and KD CUTLL1 cells determined by Annexin-V and 7-AAD staining. Data are 3 technical replicates, representative of 2 experiments. **c,** Mean fluorescence intensity (MFI) of Z-VAD-FMK, a dye for active caspase, normalized to CT values. Data are biological replicates from 3 independent experiments, n=7. Student's t-test, two-tailed. **d,** Proliferation of sorted live CT and KD CUTLL1 cells determined by trypan blue counting. Part of same data shown in Figure 1j. **e,** EdU staining of CT and KD CUTLL1 cells with quantification of cells in different phases of cell cycle. Data are 3 technical replicates and representative of 2 independent experiments. **f,** CT and KD CUTLL1 cells were analyzed by flow cytometry for TCR, CD4, and CD8 expression. The MFI of each were normalized to the FSC of the cells. Data are presented as mean ± SD of 3 biological replicates, representative of 3 experiments. Student's t-test, two-tailed, ns > 0.05. **g,** CT and KD CUTLL1 cells were cultured in the presence or absence of 5µg/ml anti-CD3 and 1µg/ml anti-CD28 for 30 minutes at 37°C. The TCR MFI was determined by flow cytometry and normalized to the unstimulated MFI. Data are 3 technical replicates. **h,** GSEA plots comparing genes that are up (top) or down (bottom) in T-ALL cells lacking the NOTCH dependent *MYC* super enhancer (N-Me) in CT and KD CUTLL1 cells. NES: normalized enrichment score, FDR: false discovery rate. The publicly available gene list used in this study is available in the Pubmed database under accession code PMID: 25194570[1]. **i,** GSEA plots comparing genes that are down-regulated by MYC (top) and up-regulated by MYC (bottom) in CT and KD CUTLL1 cells. The publicly available gene list used in this study is available in the Pubmed database under accession code PMID: 16116477[2]. **(h, i)** p-values determined by Weighted Kolmogorov-Smirnov test and adjusted for multiple comparisons. **j,** Heatmaps of DEGs from MYC-target gene and cell cycle Hallmark pathways. Source data are provided as a Source Data file.

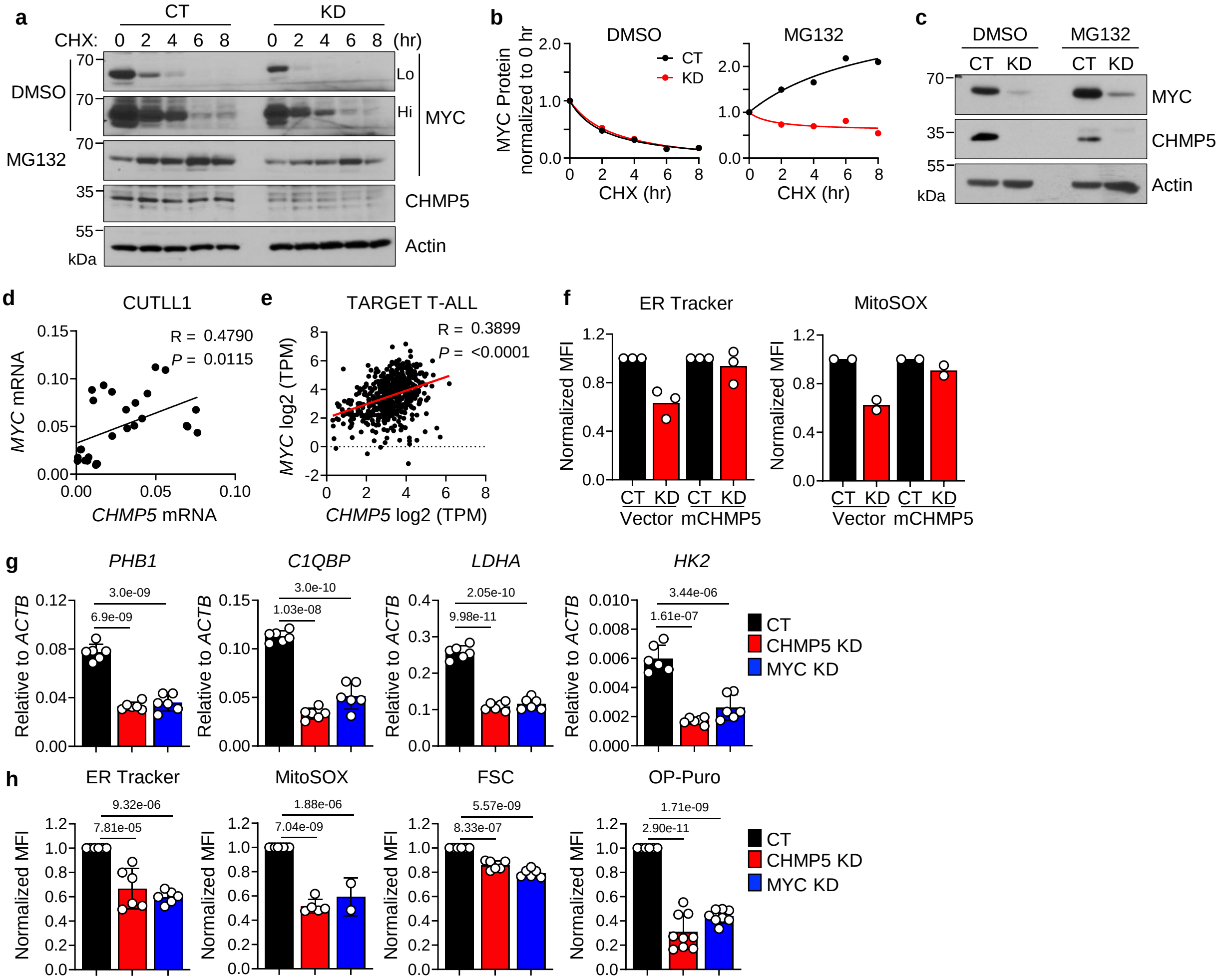

Supplementary Figure 2. CHMP5 deficiency phenocopies MYC deficiency.

a, Western blot of CUTLL1 cells treated with cycloheximide (CHX) overtime with vehicle (DMSO) or 10 µM MG132. Lo: short exposure for MYC; Hi: long exposure for MYC. **b,** Quantification of MYC expression in DMSO and MG132 treated samples from (**a**) overtime, normalized to the 0-time point of each group. Data is representative of 2 experiments. **c,** Western blot of CT and KD CUTLL1 cells treated with vehicle (DMSO) or 10 µM MG132 for 6 hours. Representative of 2 experiments. **d,** Correlation of MYC mRNA and CHMP5 mRNA (normalized to *ACTB*) in CUTLL1. Rho (R) and p-values determined by Pearson Correlation test. n=27 **e,** Correlation of MYC mRNA and CHMP5 mRNA (transcripts per million) in T-ALL patient samples from the TARGET T-ALL dataset. Rho (R) and p-values determined by Pearson Correlation test. n=532. The TARGET-TALL publicly available data used in this study are available in the NCBI database of Genotypes and Phenotypes under accession code phs000464.v7.p1[3]. **f,** Mean fluorescence intensity (MFI) of ER Tracker and MitoSOX dyes in CT and KD CUTLL1 transduced with murine CHMP5 (mCHMP5) or vector control. Data are technical replicates (n=3 ER, n=2 MitoSOX), representative of 2 experiments. **g,** mRNA expression of MYC-target metabolic genes. Data points are biological replicates from 3 independent experiments, n= 6 for all groups. One-way ANOVA. **h,** Flow analysis of CUTLL1 cells treated with shRNAs for CT, CHMP5 or MYC. MFIs are normalized to CT. Data points are biological replicates from 3 independent experiments. One-way ANOVA. ER Tracker: n=6 for all groups. MitoSOX: n=5 for CT and CHMP5-KD, n=2 for MYC-KD. FSC: n=6 for all groups. OPP: n=8 for CT and MYC-KD, n=9 for CHMP5-KD. Data are representative of 3 independent experiments. Source data are provided as a Source Data file.

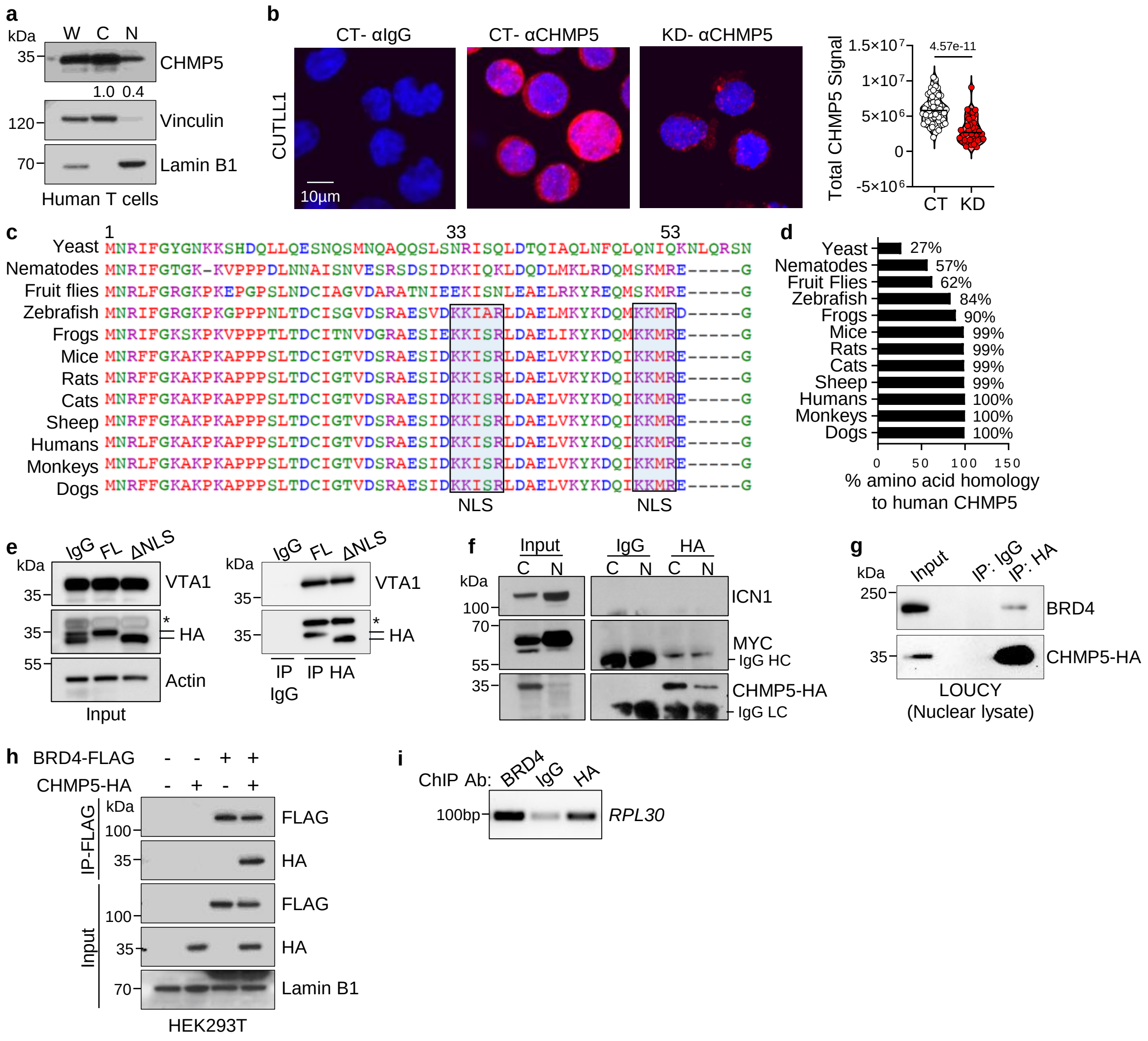

Supplementary Figure 3. Identification of nuclear CHMP5-BRD4 interaction on chromatin.
a, Western blot of fractionated human T cells isolated from PBMCs. W, whole cell; C, cytoplasmic; and N, nuclear lysates. Nuclear CHMP5 band intensity relative to the cytoplasmic band. Representative of 2 experiments. **b,** Immunofluorescence images of CT and KD CUTLL1 stained with IgG, or anti-CHMP5 primary antibodies followed by a secondary AF647 antibody (Red) and DAPI for nuclear staining (blue). Images are representative of 8 samples (4 CT, 4 KD) from 2 independent experiments. Scale bar indicates 10µm. The total signal of CHMP5 staining in CUTLL1 CT (n=68) and KD (n=41) cells is quantified. Student's t-test, two-tailed. **c,** Clustal Omega alignment of the N-terminal amino acid sequences of CHMP5 from different species. Sequences corresponding to the bipartite nuclear localization sequence (NLS) are highlighted. **d,** Amino acid sequence homology of CHMP5 from different species relative to human CHMP5. **e,** Immunoprecipitation of FL and ΔNLS CHMP5 with anti-HA from the cytoplasmic fraction of CUTLL1 cells. Immunoblotted with VTA1. * indicates the leftover VTA1 band. FL-CHMP5 is the middle band and ΔNLS CHMP5 is the lower band of the HA blot. The IgG sample was a mix of the FL and NLS lysates. **f,** Western blot of fractionated CUTLL1 cells transduced with CHMP5-HA and subjected to immunoprecipitation with IgG or anti-HA antibodies and immunoblotted for MYC and ICN1. Representative of 3 experiments. **g,** Western blot of nuclear lysates in CHMP5-HA transduced LOUCY cells subjected to immunoprecipitation with IgG or anti-HA antibodies Representative of 2 experiments. **h,** Nuclear lysate of HEK293T cells co-transfected with BRD4-FLAG or CHMP5-HA and subsequently immunoprecipitated with anti-FLAG antibodies and immunoblotted with anti-HA or anti-FLAG antibodies. Representative of 2 experiments. **i,** PCR for housekeeping gene *RPL30* from total ChIP DNA after ChIP with BRD4, IgG, or HA antibodies from CHMP5-HA CUTLL1 cells, run on a 1% agarose gel. Representative of 2 experiments. Source data are provided as a Source Data file.

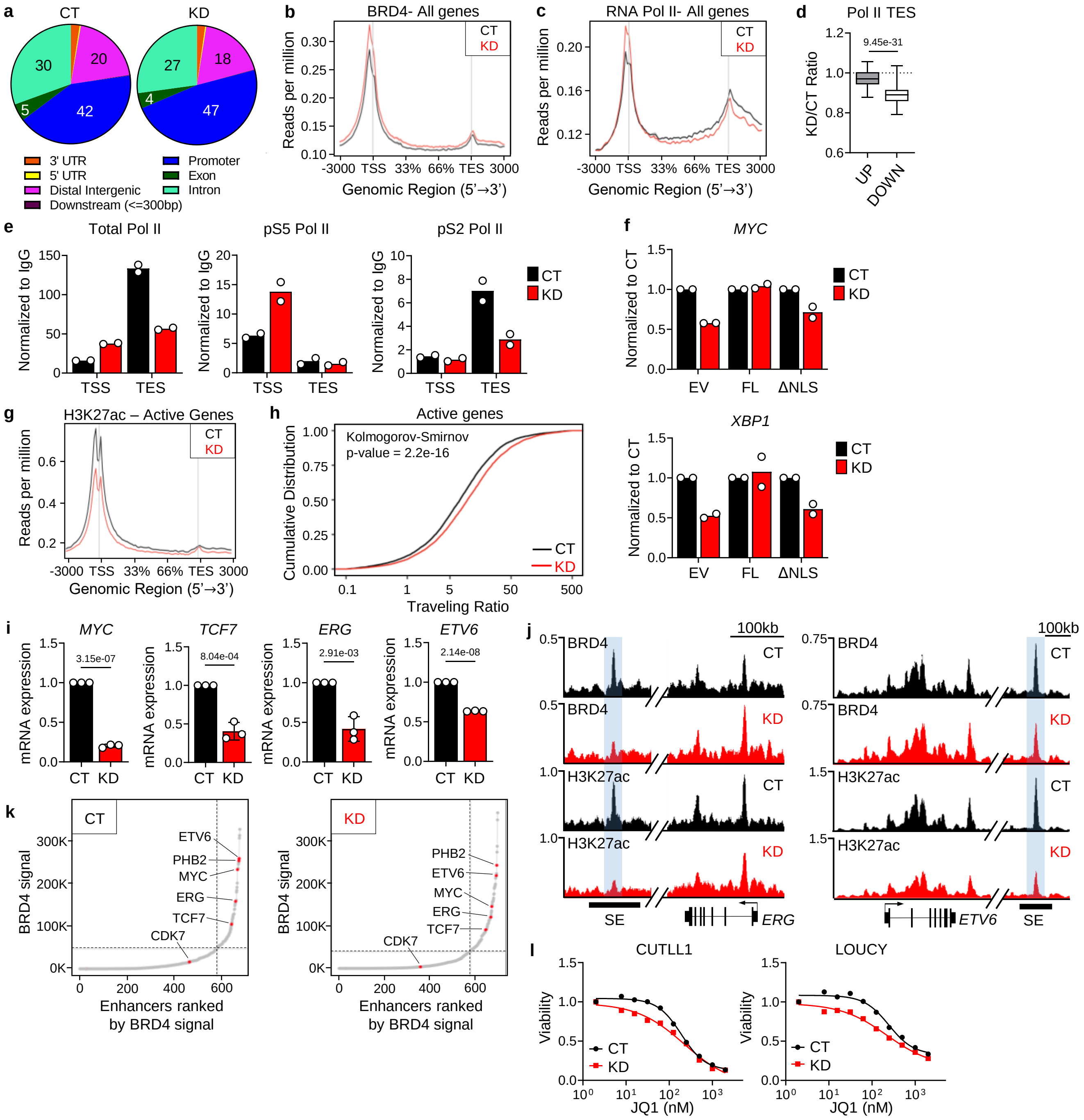
a
CT
KD
30
20
5
42
27
18
4
47
3' UTR
5' UTR
Distal Intergenic
Downstream (<=300bp)
Promoter
Exon
Intron
b
BRD4- All genes
CT
KD
Reads per million
Genomic Region (5'→3')
c
RNA Pol II- All genes
d
Pol II TES
9.45e-31
KD/CT Ratio
UP
DOWN
e
Total Pol II
pS5 Pol II
pS2 Pol II
Normalized to IgG
TSS
TES
f
MYC
XBP1
Normalized to CT
EV
FL
ΔNLS
g
H3K27ac – Active Genes
h
Active genes
Kolmogorov-Smirnov
p-value = 2.2e-16
Cumulative Distribution
Traveling Ratio
i
MYC
TCF7
ERG
ETV6
3.15e-07
8.04e-04
2.91e-03
2.14e-08
mRNA expression
j
BRD4
H3K27ac
100kb
SE
ERG
ETV6
k
BRD4 signal
ETV6
PHB2
MYC
ERG
TCF7
CDK7
Enhancers ranked
by BRD4 signal
l
CUTLL1
LOUCY
Viability
JQ1 (nM)

Supplementary Figure 4. CHMP5 mediates BRD4-driven Pol II pause release and super enhancer formation.
a, Pie chart of genome-wide BRD4 occupancy in control (CT) and CHMP5-depleted (KD) CUTLL1 cells. **b,** Metaplot of BRD4 binding at the TSS, gene body, and TES across the genome in CT and KD CUTLL1 cells. Shaded bands represent standard error (SE), data is representative of 2 biological replicates. **c,** Metaplot of Pol II binding at the TSS, gene body, and TES across the genome in CT and KD cells. Shaded bands represent standard error (SE), data is representative of 2 biological replicates. **d,** Box plot showing the ratio of KD to CT Pol II enrichment at the TES of UP- and DOWN-regulated DEGs from RNA-seq on CT and KD CUTLL1 cells (Figure 1). Boxplot limits are the 25th and 75th percentiles, with median in the middle. Whiskers extend to the minimum and maximum values, n= 101 genes per group. Student's t-test, two-tailed. **e,** ChIP-qPCR of total Pol II, phospho-serine 5 (pS5) Pol II, and phospho-serine 2 (pS2) Pol II at the TSS and TES of the *MYC* locus in CT and KD CUTLL1 cells. Data are 2 technical replicates. **f,** CUTLL1 CT and KD cells were transduced with empty vector (EV), FL, or ΔNLS mCHMP5. After 48hours, GFP+ cells were sorted for qPCR. *MYC* and *XBP1* mRNA is relative to *ACTB* and normalized to its own control sample. Data points are 2 biological replicates from two independent experiments. **g,** Metaplot of H3K27ac density at the TSS, gene body and TES across all active genes in CT and KD CUTLL1 cells. Shaded bands represent standard error (SE), data is representative of 2 biological replicates. **h,** Pol II traveling ratio at active genes (defined by H3K27ac signal at promoter). Kolmogorov-Smirnov test is two-sided and not adjusted for multiple comparisons, n=6932 genes (CT), n= 6695 genes (KD). **i,** Relative mRNA expression of *MYC, TCF7, ERG*, and *ETV6.* Values were normalized to *ACTB*, then normalize to CT cells. Data are mean ± SD of three biological replicates. Student's t-test, two-tailed. **j,** BRD4 and H3K27ac ChIP-seq tracks at the *ERG* and *ETV6* gene loci. SE, super-enhancer. **k,** Hockey stick plots of ranked genome-wide BRD4 signals in CT (left) and KD (right) CUTLL1 cells. Positions of key T-ALL genes are highlighted. **l,** MTT viability assays for CT and KD CUTLL1 and LOUCY cells treated with JQ1 for 72 hours. Data are mean ± SD of 3 technical replicates. Source data are provided as a Source Data file.

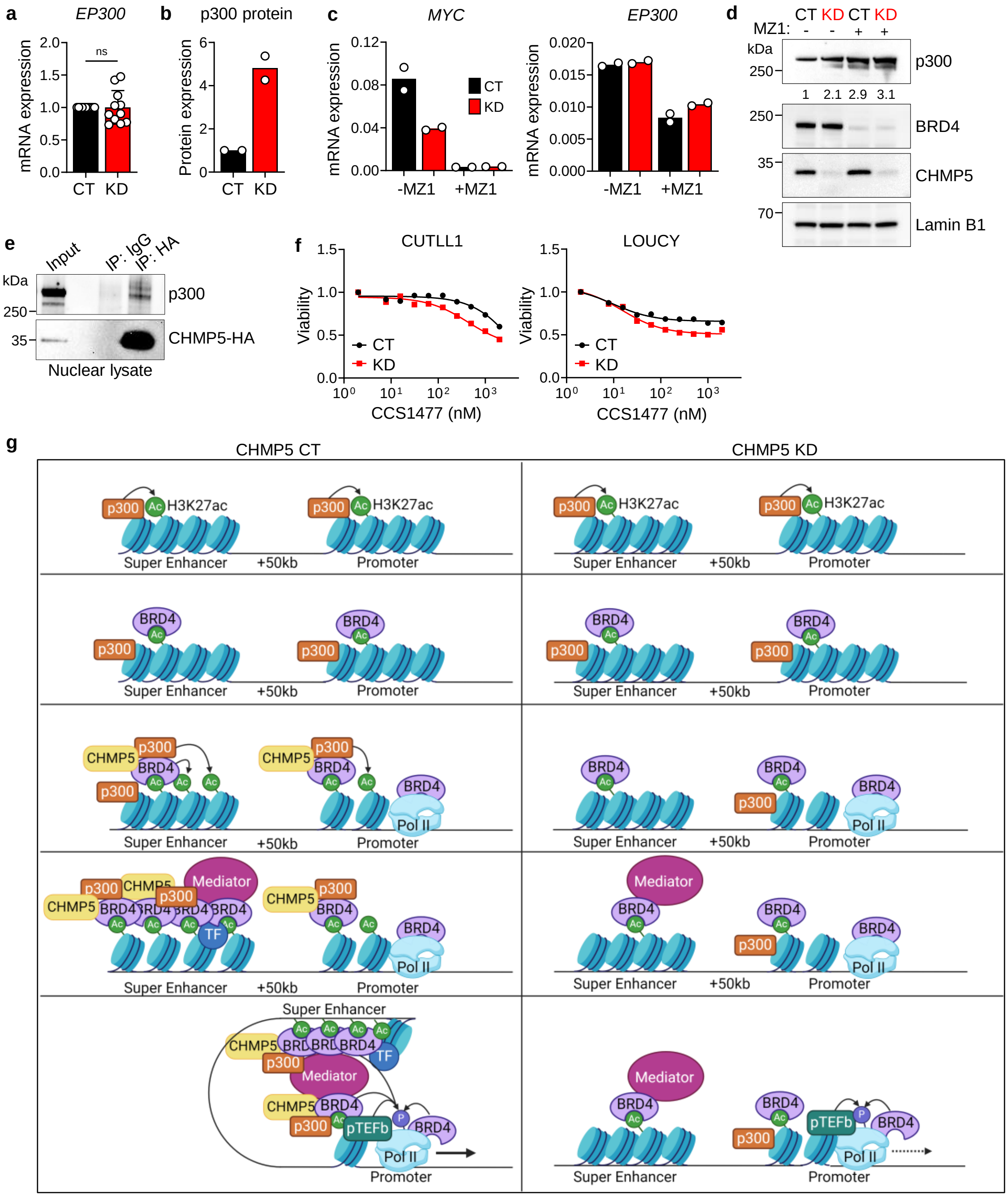

Supplementary Figure 5. CHMP5 promotes the interaction between BRD4 and p300.

a, Relative mRNA (normalized to *ACTB*) of *EP300* in CT and KD CUTLL1 cells. Data are 11 biological replicates from 3 individual experiments. Student's t-test, two-tailed, p= 0.9892. **b,** p300 protein expression (normalized to Lamin B1) in CUTLL1 cells shown in **Figure 4a**. Data are biological replicates from two independent experiments. **c-d,** qPCR of *MYC* and *EP300* **(c)**, and western blot of nuclear lysate **(d)** from CT and KD CUTLL1 cells treated with 100nM of the BRD4-PROTAC MZ1 for 4 hours, with quantification of p300 protein levels relative to CT-MZ1. Representative of 2 experiments. qPCR data are 2 technical replicates. **e,** Nuclear lysate from lentiviral CHMP5-HA-transduced CUTLL1 cells immunoprecipitated with IgG or anti-HA antibody and immunoblotted for p300. Representative of 2 experiments. **f,** MTT viability assays of CT and KD CUTLL1 and LOUCY cells treated with CCS1477 for 72 hours. Data are mean of 3 technical replicates. **g,** Mechanistic model of CHMP5-mediated regulation of epigenetic and transcriptional program in T-ALL cells. *Created in BioRender. Umphred-Wilson, K. (2025)* https://BioRender.com/7yvna84. In wildtype T-ALL cells (left), CHMP5 potentiates the p300-BRD4 interaction that mediates H3K27 hyperacetylation of *cis* enhancers and super-enhancers. Subsequent assembly of core transcriptional factors at promoters and enhancers enables proximal-promoter and distal enhancer interaction that stimulate Pol II pause-release and transcriptional elongation of pro-leukemogenic genes. By contrast, CHMP5 deficiency (right) impairs the p300-BRD4 interaction, which reduces H3K27 acetylation, and disrupts super-enhancer formation and interaction with proximal-promoters leading to impaired transcription of T-ALL genes. Source data are provided as a Source Data file.

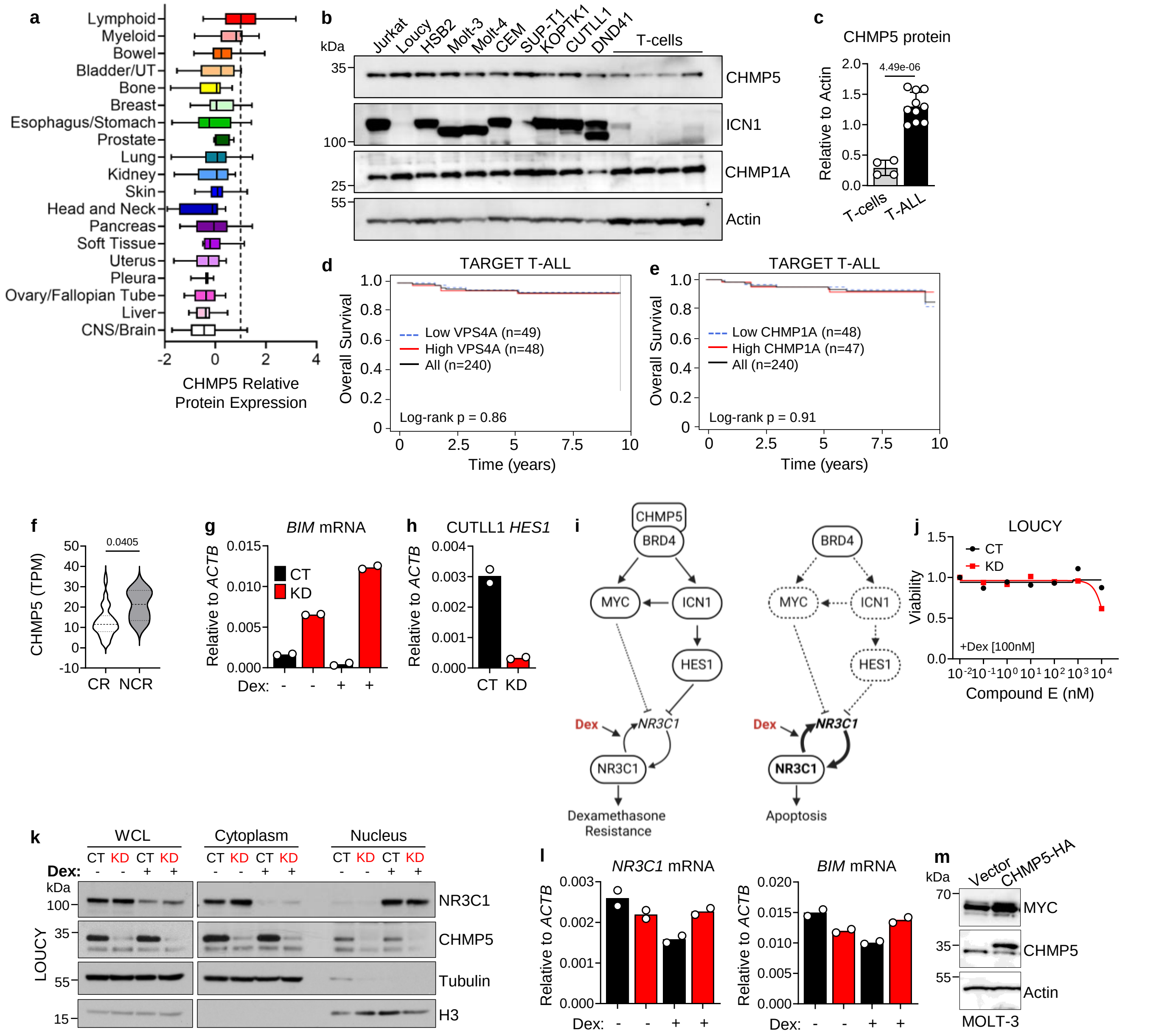

Supplementary Figure 6. High CHMP5 expression correlates with poor prognosis and promotes chemoresistance in T-ALL. a, Relative CHMP5 protein expression in cancer cell lines in order from highest to lowest average. Data from Cancer Cell Line Encyclopedia (CCLE)[4]. Boxplot limits are the 25th and 75th percentiles, with median in the middle. Whiskers extend to the minimum and maximum values. **b,** Western blot of T-ALL cell lines and healthy human T-cells. Representative of 3 experiments. **c,** Quantification of CHMP5 protein relative to Actin from (**b**). Data are mean ±SD of biological replicates, n=4 T cells, n=10 T-ALL. Student's t-test, two-tailed. **d-e,** Overall survival of pediatric T-ALL patients (TARGET T-ALL) expressing high (top 20%) and low (bottom 20%) levels of *VPS4A* **(d)** and *CHMP1A* **(e)**. Log-rank test. The TARGET-TALL publicly available data used in this study are available in the NCBI database of Genotypes and Phenotypes under accession code phs000464.v7.p1[3]. **f,** mRNA expression of *CHMP5* in pediatric T-ALL patients that achieved complete remission (CR) (n = 65) or did not achieve complete remission (NCR) (n = 4). This publicly available data used in this study are available in the National Omics Data Encyclopedia (NODE) under accession code OEP00000760[5]. **g,** Expression of *BIM* in CUTLL1 cells treated with and without 1uM dexamethasone. Data are 2 technical replicates, representative of 2 experiments. **h,** Expression of *HES1* in CT and KD CUTLL1 cells. Data are 2 technical replicates, representative of 3 experiments. **i,** Schematic of how BRD4 regulates MYC and ICN1 expression to inhibit NR3C1 transcription in CHMP5 CT (left) and KD (right) cells. *Created in BioRender. Adoro, S. (2025) https://BioRender.com/y0d31ws.* **j,** Viability of CT and KD LOUCY cells treated with Compound E (Comp E) plus 100 nM dexamethasone (Dex) for 3 days. Data are presented as mean of 3 technical replicates. Representative of 2 experiments. **k,** Western blot of fractionated CT and KD LOUCY cells treated with vehicle (DMSO) or 1 μM dexamethasone (Dex) for 18 hours. Representative of 2 experiments. **l,** Expression of *NR3C1* and *BIM* in LOUCY cells from (**k**). Data are 2 technical replicates. Representative of 2 experiments. **m,** Western blot of MOLT-3 cells transduced with empty vector or CHMP5-HA lentivirus. Representative of 2 experiments. Source data are provided as a Source Data file.

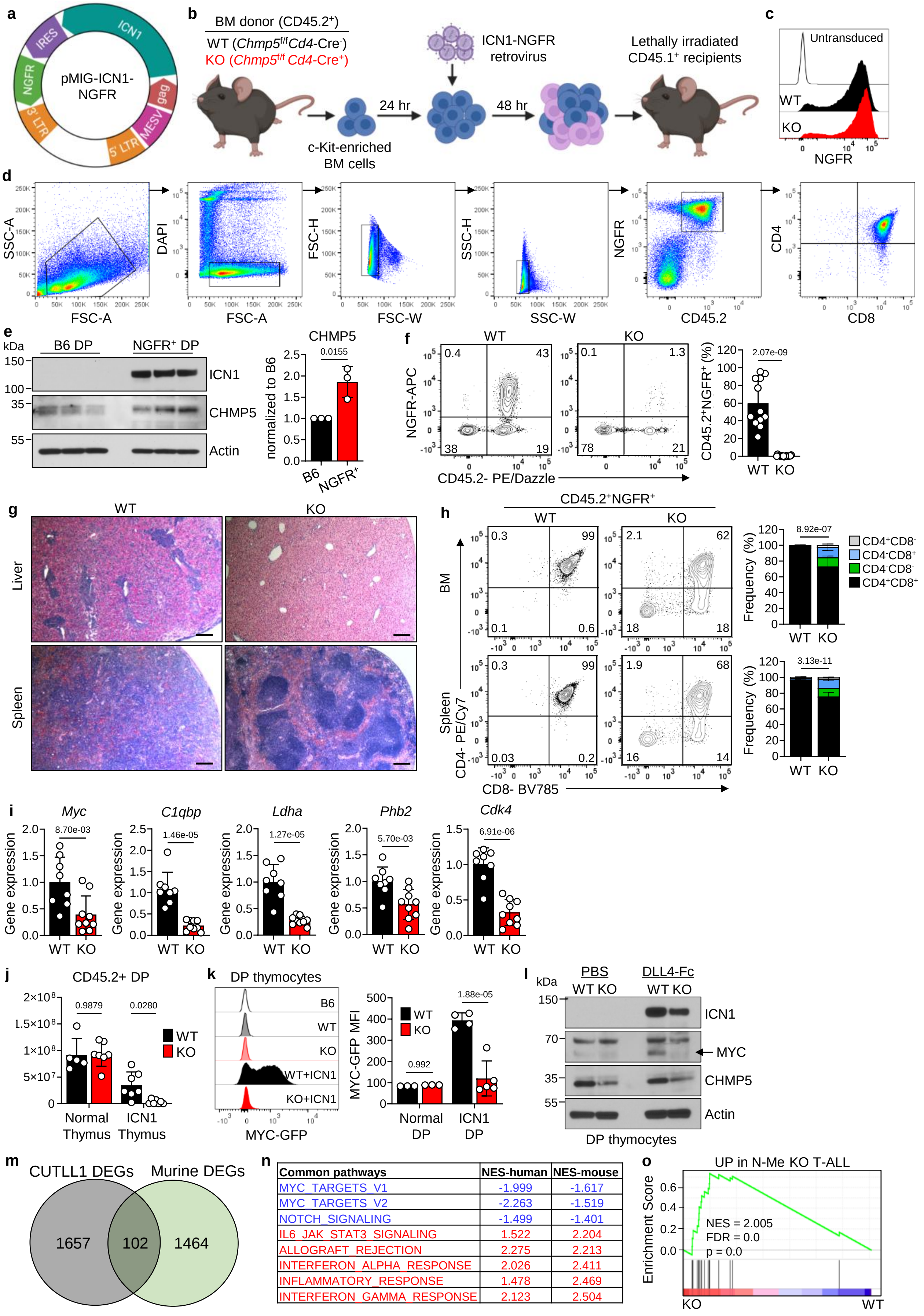
a
pMIG-ICN1-NGFR
ICN1
IRES
NGFR
3' LTR
5' LTR
MESV
gag
b
BM donor (CD45.2+)
WT (Chmp5f/f Cd4-Cre-)
KO (Chmp5f/f Cd4-Cre+)
24 hr
c-Kit-enriched BM cells
ICN1-NGFR retrovirus
48 hr
Lethally irradiated CD45.1+ recipients
c
Untransduced
WT
KO
NGFR
d
SSC-A
FSC-A
DAPI
FSC-H
FSC-W
SSC-H
SSC-W
NGFR
CD45.2
CD4
CD8
e
kDa
B6 DP
NGFR+ DP
ICN1
CHMP5
Actin
CHMP5
normalized to B6
0.0155
B6
NGFR+
f
WT
KO
NGFR-APC
CD45.2- PE/Dazzle
CD45.2+NGFR+ (%)
2.07e-09
WT KO
g
WT
KO
Liver
Spleen
h
CD45.2+NGFR+
WT
KO
BM
Spleen
CD4- PE/Cy7
CD8- BV785
Frequency (%)
8.92e-07
3.13e-11
CD4+CD8-
CD4-CD8+
CD4-CD8-
CD4+CD8+
WT KO
i
Myc
C1qbp
Ldha
Phb2
Cdk4
Gene expression
8.70e-03
1.46e-05
1.27e-05
5.70e-03
6.91e-06
WT KO
j
CD45.2+ DP
0.9879
0.0280
WT
KO
Normal Thymus
ICN1 Thymus
k
DP thymocytes
B6
WT
KO
WT+ICN1
KO+ICN1
MYC-GFP
MYC-GFP MFI
0.992
1.88e-05
Normal DP
ICN1 DP
l
kDa
PBS
DLL4-Fc
WT KO
ICN1
MYC
CHMP5
Actin
DP thymocytes
m
CUTLL1 DEGs
Murine DEGs
1657
102
1464
n
Common pathways | NES-human | NES-mouse
MYC_TARGETS_V1 | -1.999 | -1.617
MYC_TARGETS_V2 | -2.263 | -1.519
NOTCH_SIGNALING | -1.499 | -1.401
IL6_JAK_STAT3_SIGNALING | 1.522 | 2.204
ALLOGRAFT_REJECTION | 2.275 | 2.213
INTERFERON_ALPHA_RESPONSE | 2.026 | 2.411
INFLAMMATORY_RESPONSE | 1.478 | 2.469
INTERFERON_GAMMA_RESPONSE | 2.123 | 2.504
o
UP in N-Me KO T-ALL
Enrichment Score
NES = 2.005
FDR = 0.0
p = 0.0
KO
WT

Supplementary Figure 7. CHMP5 deficiency impairs T-ALL development and progression in vivo.
a, Plasmid map of bicistronic ICN1 and NGFR expression retroviral plasmid. IRES, internal ribosomal entry site. *Created in BioRender. Umphred-Wilson, K. (2025) https://BioRender.com/zwr0vwm*. **b,** Retrovirus-induced ICN1 leukemia mice experimental scheme. *Created in BioRender. Adoro, S. (2025) https://BioRender.com/iv9tesj*. **c,** NGFR expression 48 hours after transduction of WT and KO donor BM cells. **d,** Gating strategy for flow cytometry analysis. Lymphocytes—live cells—singlets—CD45.2^{+}NGFR^{+}—CD4^{+}CD8^{+}. **e,** CHMP5 expression in sorted CD4/CD8 double positive (DP) cells from the thymus of n=3 B6 mice and the spleen of n=3 WT NGFR^{+} mice. (right) Quantification of CHMP5 relative to actin and normalized to B6 samples. Each point is a biological replicate. P values, Student's t-test, two-tailed. Data are representative of 2 independent experiments with 3 mice/group. **f,** Representative flow cytometry analysis of blood from leukemia mice at 4 weeks post-transplant with mean (± SD) frequency of CD45.2^{+}NGFR^{+} cells shown in graph. Student's t-test, two-tailed. WT n=12, KO n=15 mice/group. **g,** Hematoxylin and eosin staining of liver and spleen from leukemia mice. Scale bar = 200 µm. Representative of 15 samples from 3 independent experiments. **h,** Flow cytometry plots of CD4 and CD8 expression on CD45.2^{+}NGFR^{+} cells with mean frequency ±SD of gated subsets. BM: WT, n=4 mice, KO, n=5 mice. Spleen: n=4 mice/group. 2-way ANOVA. **i,** mRNA expression of *Myc, C1qbp, Ldha, Phb2, Cdk4* in splenic CD45.2^{+}NGFR^{+} cells from WT (n = 8) and KO (n = 9) chimera mice. Expression is normalized to WT and presented as mean ±SD. Student's t-test, two-tailed. **j,** Number of DP cells in the thymus of normal Chmp5 CD4-cre WT and KO mice and ICN1-induced WT and KO leukemic mice. n=5 (normal WT), n=7 (normal KO, ICN WT, ICN KO). 2-way ANOVA. **k,** MYC-GFP expression in DP cells from the thymus of WT and KO normal and leukemic mice, with quantification of the MFI. n=3 Normal DP samples, n=4 WT ICN1-DP, n=5 KO ICN1-DP. **l,** DP thymocytes sorted from the thymus of normal *Chmp5*$^{f/f}$*Cd4*-Cre^{-} (WT) or *Chmp5*$^{f/f}$*Cd4*-Cre^{+} (KO) mice and cultured with or without plate bound DLL4 (5 µg/ml) overnight and harvested for western. Representative of 2 experiments. **m,** Venn-diagram of DEGs overlapping from CT and KD CUTLL1 (**Figure 1**), and WT and KO NGFR^{+} splenocytes. **n,** Overlapping differentially expressed pathways from CT and KD CUTLL1, and WT and KO NGFR^{+} splenocytes. **o,** GSEA plots comparing genes that are up in T-ALL cells lacking the NOTCH dependent *MYC* super enhancer (N-Me) in CT and KD cells. The publicly available gene list used in this study are available in the Pubmed database under accession code PMID: 25194570[1]. p-value determined by Weighted Kolmogorov-Smirnov test and adjusted for multiple comparisons. Source data are provided as a Source Data file.

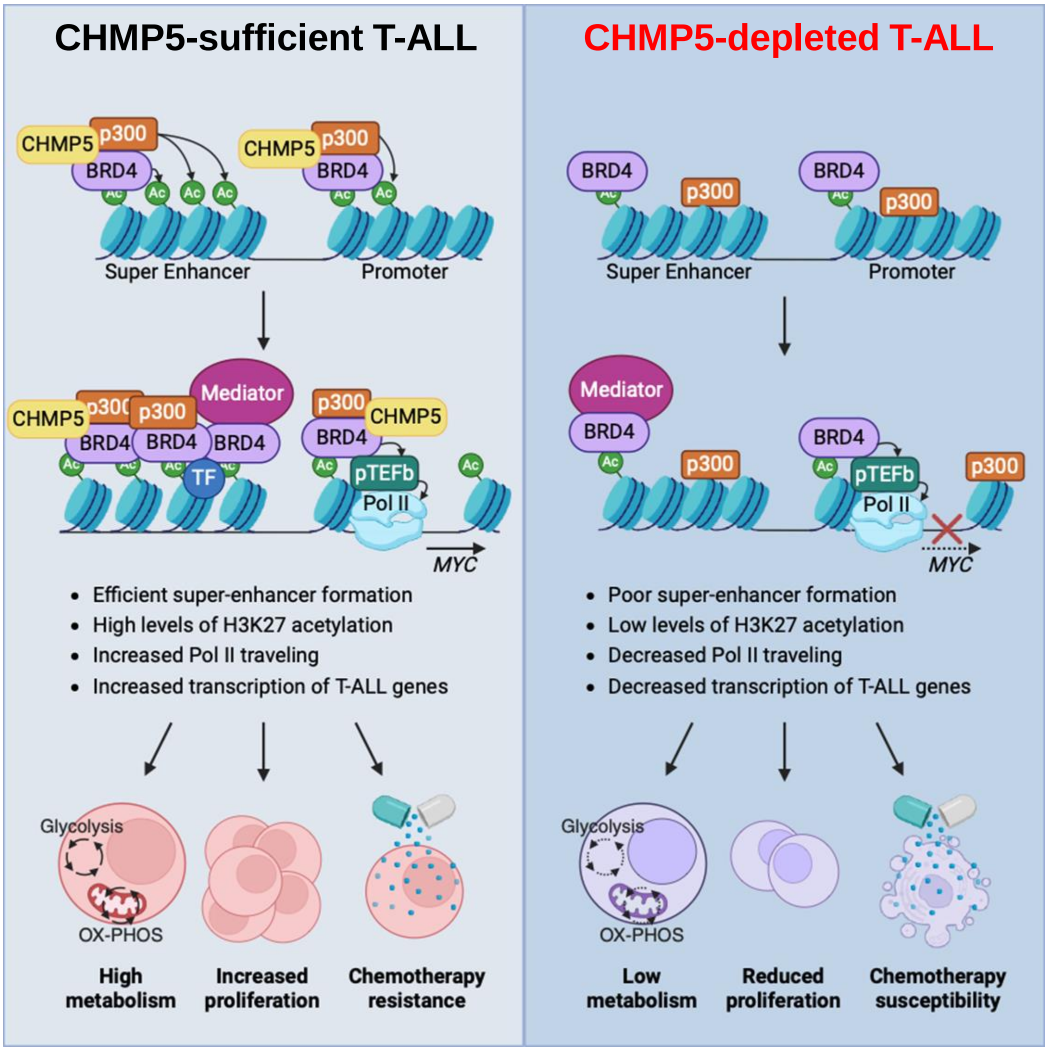

Supplementary Figure 8. CHMP5 promotes T-ALL by enabling BRD4-p300 dependent transcription. In CHMP5-sufficient T-ALL (left), CHMP5 promotes p300 and BR4 activity to drive the transcription of key T-ALL genes, especially *MYC*. Subsequently, transcription of these genes enhances T-ALL metabolism, proliferation, and chemoresistance. Thus, high expression of CHMP5 correlates with poorer survival in T-ALL patients. In CHMP5-deficient T-ALL (right), p300 and BRD4 activity is limited, reducing the transcription of T-ALL leukemic genes. Therefore, CHMP5-deficient T-ALL cells have reduced metabolic activity, proliferation, and chemoresistance. When CHMP5 is absent during the development of T-ALL *in vivo*, mice fail to develop disease. *Created in BioRender. Umphred-Wilson, K. (2025) https://BioRender.com/g8dus2w*.

Sample	Status at collection	Treatment	Active NOTCH1	Phenotypic markers
P1	Relapsed	Hydrea, leukopheresis	Yes	CD38+, CD34+ subset, CD3 int, CD5 dim, HLA-DR+ subset
P2	De Novo	Hydrea, rasburicase	Yes	CD38+. CD34-, CD3+, CD5+, HLA-DR int
P3	De Novo	Hydrea	Yes	CD38+, CD34+, CD3+, HLA-DR dim
P4	De Novo	Allopurinol, leukopheresis	Yes	CD38+, CD34-, CD3-, CD5-, HLA-DR-

Supplementary Table 1. Patient information for primary T-ALL samples

Antibody	Source	Catalog number	Application	Dilution
CHMP5	Thermo Fisher Scientific	PA563303	WB, IF	1:1000, 2ug/ml
CHMP1A	Proteintech	15761-1-AP	WB	1:1000
VPS4A (A-11)	Santa Cruz Biotechnology	sc-393428	WB	1:250
VTA1 (Polyclonal)	Proteintech	24-041863	WB	1:1000
MYC (Polyclonal)	Thermo Scientific	10828-1-AP	WB	1:1000
MYC (D84C12) rabbit	Cell Signaling Technology	5605S	WB	1:1000
MYC (C33) mouse	Santa Cruz Biotechnology	sc-42	WB	1:500
BRD4 rabbit	Bethyl Laboratories	A301-985A50	WB, IP	1:1000
BRD4 (A-7) mouse	Santa Cruz Biotechnology	sc-518021	WB	1:500
Bcl-2	Cell Signaling Technology	2498S	WB	1:1000
Cleaved Notch1 (Val1744) (D3B8)	Cell Signaling Technology	4147S	WB	1:1000
Pol II (8WG16)	Santa Cruz Biotechnology	sc-56767	WB	1:1000
MED1	Abcam	ab64965	WB	1:1000
P300 (F-4)	Santa Cruz Biotechnology	sc-48343	WB	1:500
NR3C1 (D8H2)	Cell Signaling Technology	3660T	WB	1:1000
β-Actin (D6A8)	Cell Signaling Technology	8457S	WB	1:2000
Lamin B1 (D4Q4Z)	Cell Signaling Technology	12586S	WB	1:1000
Vinculin	Cell Signaling Technology	4650S	WB	1:1000
Tubulin	Cell Signaling Technology	2144S	WB	1:1000
Histone H3	Cell Signaling Technology	4499S	WB	1:5000
FLAG-Tag (M2) Mouse	Sigma-Aldrich	F1804	WB, IP	1:2000
FLAG-Tag (D6W5B) Rabbit	Cell Signaling Technology	14793S	WB	1:1000
HA-Tag (C29F4) Rabbit	Cell Signaling Technology	3724S	WB, IP	1:2000
HA-Tag (6E2) Mouse	Cell Signaling Technology	2367S	WB	1:1000
Anti-mouse IgG, HRP linked Antibody	Cell Signaling Technology	7076S	WB	1:5000
Anti-rabbit IgG, HRP linked Antibody	Cell Signaling Technology	7074S	WB	1:5000
RNA pol II	Active Motif	39097	ChIP	4ug
Histone H3K27ac	Active Motif	39085	ChIP	4ug
BRD4 (AbFlex)	Active Motif	91301	ChIP	4ug
P300	Active Motif	61401	ChIP	4ug
HA-Tag	Abcam	ab9110	ChIP	4ug
Rabbit IgG	Cell Signaling Technology	2729S	ChIP, IF, IP	4ug, 2ug/ml
RNA Pol II CTD pS2	Active Motif	91115	ChIP	4ug
RNA Pol II CTD pS5	Cell Signaling Technology	13523	ChIP	4ug
Annexin V APC	BioLegend	640941	FC	1:25
Human NGFR APC (ME20.4)	BioLegend	345108	FC	1:100
Mouse CD45.1 BV421 (A20)	BioLegend	110732	FC	1:100
Mouse CD45.2 APC/Fire750 (104)	BioLegend	109852	FC	1:100
Mouse CD45.2 PE/Dazzle (104)	BioLegend	109846	FC	1:100
Mouse CD4 PE/Cy7 (GK1.5)	BioLegend	100422	FC	1:100
Mouse CD8 BV786 (53-6.7)	BioLegend	100750	FC	1:100
Mouse CD34 AF700 (RAM34)	BD Biosciences	560518	FC	1:100
Mouse CD19 BV605 (6D5)	BioLegend	115540	FC	1:100
Mouse CD3 AF700 (17A2)	BioLegend	100216	FC	1:100

Supplementary Table 2. Antibodies used for experiments
WB: western blot, IP: immunoprecipitation, ChIP: Chromatin immunoprecipitation, IF: immunofluorescence, FC: flow cytometry.

Name	Target	Target Sequence	Catalog number
CT1	Non-targeting	GCGCGATAGCGCTAATAATTT	SHC016 (Sigma)
CT2	Non-targeting	CCTAAGGTTAAGTCGCCCTCG	136035 (Addgene)
KD1	CHMP5	GAATCCATTGACAAGAAGATT	TRCN0000159946
KD2	CHMP5	GAGTTGGATGCACTAGGTGAT	TRCN0000163206
MYC KD1	MYC	CAGTTGAAACACAAACTTGAA	TRCN0000039640
MYC KD2	MYC	CCCAAGGTAGTTATCCTTAAA	TRCN0000039639

Supplementary Table 3. Target sequences for shRNAs

**Gene**	**Species**	**forward**	**reverse**	**Application**
ACTB	human	GCGAGAAGATGACCCAGATC	CCAGTGGTACGGCCAGAGG	RT-qPCR
CHMP5	human	TGATGAGCTTCTGGCTGATG	TCTGTGGCAATCCAAATTCA	RT-qPCR
MYC	human	GCTGCTTAGACGCTGGATTT	CGAGGTCATAGTTCCTGTTGG	RT-qPCR
PHB1	human	GCGTGGTGAACTCTGCCTTA	TGTACCCACGGGATGAGAAA	RT-qPCR
C1QBP	human	ATTAGTGCGGAAAGTTGCCGGGG	GCTCCTGTTCTTCAACCTTCTGCC	RT-qPCR
LDHA	human	TTGACCTACGTGGCTTGGAAG	GGTAACGGAATCGGGCTGAAT	RT-qPCR
HK2	human	AAGGCTTCAAGGCATCTG	CCACAGGTCATCATAGTTCC	RT-qPCR
NR3C1	human	GGCAATACCAGGTTTCAGGA	ACACAGCAGGTTTGCACTTG	RT-qPCR
BIM	human	CAGATATGCGCCCAGAGATA	ACCAGGCGGACAATGTAAC	RT-qPCR
NOTCH1	human	CACTGTGGGCGGGTCC	GTTGTATTGGTTCGGCACCAT	RT-qPCR
HES1	human	AGGCGGACATTCTGGAAATG	CGGTACTTCCCCAGCACACTT	RT-qPCR
XBP1	human	AGGAGTTAAGACAGCGCTTGGGGATGG	CTGAATCTGAAGAGTCAATACCGCCAG	RT-qPCR
TCF7	human	TGCACATGCAGCTATACCCAG	TGGTGGATTCTTGGTGCTTTTC	RT-qPCR
ERG	human	CATCTCCTTCCACAGTGCCCA	CTGGATTTGCAAGGCGGCTAC	RT-qPCR
EP300	human	GATGACCCTTCCCAGCCTCAAA	GCCAGATGATCTCATGGTGAAGG	RT-qPCR
Actb	mouse	CATTGCTGACAGGATGCAGAAGG	TGCTGGAAGGTGGACAGTGAGG	RT-qPCR
Chmp5	mouse	ATGAGAGAGGGTCCTGCTAAG	CCGTGGTCTTGGTGTCCTTTA	RT-qPCR
Myc	mouse	ATGCCCCTCAACGTGAACTTC	CGCAACATAGGATGGAGAGCA	RT-qPCR
Ldha	mouse	TTCAGCGCGGTTCCGTTAC	CCGGCAACATTCACACCAC	RT-qPCR
Phb2	mouse	ACCGTGGAAGGCGGTCATA	GGTCTGGCCCGAATGTCATAG	RT-qPCR
Cdk4	mouse	AAGGTCACCCTAGTGTTTGAGC	CCGCTTAGAAACTGACGCATTAG	RT-qPCR
A- MYC enhancer	human	AAGGCCTGGAGGCAGGAGTAATTT	AGTTTGCAGCTCAGCGTTCAAGTG	CHIP-qPCR
B- MYC promoter	human	TACTCACAGGACAAGGATGCGGTT	TGAATTAACTACGCGCGCCTACCA	CHIP-qPCR
C- NDME	human	GCTGCCACATGCTGATGAAC	GCAGTTCTTCCTACGCTGGT	CHIP-qPCR
D- BDME	human	AGGAAGTGGCTTTCACATGC	GCGTGCAAAAGAGAGAAACC	CHIP-qPCR
MYC exon 1	human	ACTGGAACTTACAACACCCGAGCA	TGGACTTCGGTGCTTACCTGGTTT	CHIP-qPCR
MYC TSS	human	ACTCACAGGACAAGGATGCG	TGGACTTCGGTGCTTACCTG	ChIP-qPCR
MYC TES	human	TTCCTCTGTTGAAATGGGTCTGGG	ACCTGCCTTCTGCCATTCCTTCTA	ChIP-qPCR

Supplementary Table 4. qPCR primers used for experiments

Supplementary References:

1. Herranz, Daniel et al. “A NOTCH1-driven MYC enhancer promotes T cell development, transformation and acute lymphoblastic leukemia.” *Nature medicine* vol. 20,10 (2014): 1130-7. doi:10.1038/nm.3665
2. Kim, Y., Girard, L., Giacomini, C. et al. Combined microarray analysis of small cell lung cancer reveals altered apoptotic balance and distinct expression signatures of MYC family gene amplification. *Oncogene* 25, 130–138 (2006). https://doi.org/10.1038/sj.onc.1208997
3. Mullighan, C. G. The molecular genetic makeup of acute lymphoblastic leukemia. *Hematology Am Soc Hematol Educ Program* **2012**, 389-396 (2012). https://doi.org/10.1182/asheducation-2012.1.389
4. Barretina, J., Caponigro, G., Stransky, N. et al. The Cancer Cell Line Encyclopedia enables predictive modelling of anticancer drug sensitivity. *Nature* **483**, 603–607 (2012). https://doi.org/10.1038/nature11003
5. Chen, B. *et al.* Identification of fusion genes and characterization of transcriptome features in T-cell acute lymphoblastic leukemia. *Proceedings of the National Academy of Sciences* **115**, 373-378 (2018). https://doi.org/doi:10.1073/pnas.1717125115
