# Supplementary material for: The ESCRT protein CHMP5 promotes T cell leukemia by enabling BRD4-p300-dependent transcription
Source: Nat Commun. 2025 May 3;16:4133. doi: 10.1038/s41467-025-59504-9 (PMC12049546; doi:10.1038/s41467-025-59504-9)
Supplement: Supplementary file 2 — Reporting Summary [file 41467_2025_59504_MOESM2_ESM.pdf]

nature portfolio

Corresponding author(s): Stanley Adoro
Last updated by author(s): Apr 10, 2025

Reporting Summary

Nature Portfolio wishes to improve the reproducibility of the work that we publish. This form provides structure for consistency and transparency in reporting. For further information on Nature Portfolio policies, see our Editorial Policies and the Editorial Policy Checklist.

Statistics

For all statistical analyses, confirm that the following items are present in the figure legend, table legend, main text, or Methods section.

n/a	Confirmed	
☐	☒	The exact sample size (*n*) for each experimental group/condition, given as a discrete number and unit of measurement
☐	☒	A statement on whether measurements were taken from distinct samples or whether the same sample was measured repeatedly
☐	☒	The statistical test(s) used AND whether they are one- or two-sided<br>*Only common tests should be described solely by name; describe more complex techniques in the Methods section.*
☒	☐	A description of all covariates tested
☐	☒	A description of any assumptions or corrections, such as tests of normality and adjustment for multiple comparisons
☐	☒	A full description of the statistical parameters including central tendency (e.g. means) or other basic estimates (e.g. regression coefficient) AND variation (e.g. standard deviation) or associated estimates of uncertainty (e.g. confidence intervals)
☐	☒	For null hypothesis testing, the test statistic (e.g. *F*, *t*, *r*) with confidence intervals, effect sizes, degrees of freedom and *P* value noted<br>*Give P values as exact values whenever suitable.*
☒	☐	For Bayesian analysis, information on the choice of priors and Markov chain Monte Carlo settings
☒	☐	For hierarchical and complex designs, identification of the appropriate level for tests and full reporting of outcomes
☐	☒	Estimates of effect sizes (e.g. Cohen's *d*, Pearson's *r*), indicating how they were calculated

Our web collection on statistics for biologists contains articles on many of the points above.

Software and code

Policy information about availability of computer code

Data collection	QuantStudio six Flex real-time PCR system for RT-qPCR and ChIP-qPCR<br>BioRad iMark Microplate reader and SpectraMax ID3 for reading absorbance for BCA and MTT assays<br>BD LSR Fortessa and BD LSR-II for flow cytometry acquisition<br>Konica Minolta SRX-101a for western blot film development<br>Analytik Jena UVP ChemStudio and BioRad ChemiDoc MP for western blot digital imaging<br>XF96 Extracellular Flux Analyzer (Agilent Technologies) for Seahorse assays<br>Olympus IX73 microscope for histological images<br>Illumina HiSeq X Ten for RNA sequencing (CUTLL1)<br>Illumina NovaSeq 6000 for RNA sequencing (murine) and ChiP sequencing<br>Nikon Eclipse Ti2 microscope (IF images)
Data analysis	GraphPad Prism for data analysis and statistical tests<br>FlowJo for flow cytometry analysis<br>ImageJ for western blot quantification and IF analysis<br>Star aligner (v2.7.9a) for RNA sequencing alignment<br>htseq (v0.11.4) to generate gene counts for RNAseq<br>DEseq2 (v1.38.3) for differential expression analysis<br>GSEA 4.3.2 and MsigDB Hallmark gene set for pathway analysis<br>BioMart R and Ensembl release 105 for comparing human and mouse DEGs

April 2023

Illumina's bcl2fastq 2.17 for conversion of raw sequence files into fastq files
Bowtie2 (v2.4.5) for ChIP sequencing alignment
MACS2 (v2.7.1) for peak calling of ChIP-seq data
ChIPseeker (v1.34.1) for peak annotation
ngsplot for ChIP-seq metaplots
UCSC genome browser for ChIP track generation
ROSE software for enhancer mapping
PIC software for traveling ratio calculation
R-package 'Survival' and 'Survminer' for survival curves
Biorender for schematic generation
Clustal Omega for CHMP5 amino acid sequence alignment

For manuscripts utilizing custom algorithms or software that are central to the research but not yet described in published literature, software must be made available to editors and reviewers. We strongly encourage code deposition in a community repository (e.g. GitHub). See the Nature Portfolio guidelines for submitting code & software for further information.

Data

Policy information about availability of data

All manuscripts must include a data availability statement. This statement should provide the following information, where applicable:

- Accession codes, unique identifiers, or web links for publicly available datasets
- A description of any restrictions on data availability
- For clinical datasets or third party data, please ensure that the statement adheres to our policy

All sequencing data have been deposited to the Gene Expression Omnibus database (GEO) and are publicly available under the SuperSeries GSE244200. RNA-seq on control (CT) and shRNA-mediated CHMP5 depletion (KD) CUTLL1 human T-ALL cells can be downloaded under GEO accession number GSE244198. ChIP-seq of BRD4, Pol II, and H3K27ac in CT and KD CUTLL1 cells can be downloaded under GEO number GSE244197. Murine RNA-seq on wildtype and Chmp5-deficient ICN1-transduced CD45.2+NGFR+ splenocytes can be downloaded under GEO number GSE244199. The primary human T cells and human T-ALL54,55 datasets used in this study are available in GEO database under accession numbers GSE33470 and GSE33469 . The TARGET T-ALL data used in this study is based upon data generated by the Therapeutically Applicable Research to Generate Effective Treatments (TARGET) (https://www.cancer.gov/ccg/research/genome-sequencing/target) initiative, and is publicly available in the NCBI database of Genotypes and Phenotypes under accession code phs000464.v7.p156.. The BRD4 ChIP-seq used in Figure 2o is publicly available data available in the NCBI Gene Expression Omnibus database under accession code GSE5180041. Gene lists used for GSEA plots reported in Supplementary Figure 1h and 6o are available in the Pubmed database under accession code PMID: 25194570. Gene lists used for GSEA plots reported in Supplementary Figure 1i are available in the Pubmed database under accession code PMID: 16116477. Pediatric T-ALL patient publicly available data used in Supplementary figure 6f are available in the National Omics Data Encyclopedia (NODE) under accession code OEP00000760. The remaining data are available within the Article, Supplementary Information or Source Data file.

Software codes for quantifying immunofluorescence images are available at https://github.com/janwisn/Intensity-measurement-in-3D-segmented-cells

Research involving human participants, their data, or biological material

Policy information about studies with human participants or human data. See also policy information about sex, gender (identity/presentation), and sexual orientation and race, ethnicity and racism.

Reporting on sex and gender	Information on patient sex has not been included. In vitro data was recapitulated in cell lines and mice from both males and females.
Reporting on race, ethnicity, or other socially relevant groupings	Race, ethnicity or other socially relevant groupings were not acquired from these patients.
Population characteristics	P1: Status at collection: Relapsed. Treatment: Hydrea, leukopheresis. Genotype: N/A<br>P2: Status at collection: De Novo. Treatment: Hydrea, rasburicase. Genotype: N/A<br>P3: Status at collection: De Novo. Treatment: Hydrea. Genotype: TPMT p.Y240C, PTEN pY240C, p.P246Lfs*2, p.P246_L247insRYP, p.R233Gfs*10, p.R233*, p.R233_R234delinsLNIDHIKYRPS, p.T232_K237delinsSLQ, NOTCH1 p.L1678P<br>P4 Status at collection: De Novo. Treatment: Allopurinol, leukopheresis<br>Diagnosis for all is precursor T cell- acute lymphoblastic leukemia.<br>No age was recorded.
Recruitment	De-identified patient primary T-ALL samples were obtained from the Stem Cell and Xenograft Core at the University of Pennsylvania. All samples were collected from patients after written informed consent according to protocols approved by the University of Pennsylvania IRB (UPENN IRB 703185). De-identified patient blood was obtained from the Hematopoietic Biorepository & Cellular Therapy Core at Case Western Reserve University.
Ethics oversight	University of Pennsylvania and Case Western Reserve University

Note that full information on the approval of the study protocol must also be provided in the manuscript.

Field-specific reporting

Life sciences study design

All studies must disclose on these points even when the disclosure is negative.

Sample size	No statistical method was used to estimate sample size. Sample sizes for mouse experiments were chosen based on previous experiments and publications. Experiments were performed with at least 5 mice per group and were repeated 3 times with similar results. For in vitro experiments, assays were done with 2-3 technical replicates or with 2-3 biological replicates when indicated, and repeated 2-3 times. Sample size and replicate numbers are indicated in the figure legends.
Data exclusions	No data was excluded from these analyses.
Replication	All experiments were repeated 2 or more times (number is indicated in the figure legends) and performed in different ways (ex. immunoprecipitating in both directions) to confirm results. Data that was not able to be reproduced was not included in the paper.
Randomization	Recipient mice were housed together, irradiated, and randomly split into 2 groups for WT and KO bone marrow.
Blinding	For in vitro experiments, investigators were not blinded as they set up and analyzed all experiments themselves. For mouse studies, investigators analyzed mice by ear tag number, not group, to be unbiased in their assessments.

Reporting for specific materials, systems and methods

We require information from authors about some types of materials, experimental systems and methods used in many studies. Here, indicate whether each material, system or method listed is relevant to your study. If you are not sure if a list item applies to your research, read the appropriate section before selecting a response.

Materials & experimental systems

n/a	Involved in the study	
☐	☒	Antibodies
☐	☒	Eukaryotic cell lines
☒	☐	Palaeontology and archaeology
☐	☒	Animals and other organisms
☒	☐	Clinical data
☒	☐	Dual use research of concern
☒	☐	Plants

Methods

n/a	Involved in the study	
☐	☒	ChIP-seq
☐	☒	Flow cytometry
☒	☐	MRI-based neuroimaging

Antibodies

Antibodies used

CHMP5 Thermo Fisher Scientific PA563303, 1:1000
CHMP1A Proteintech 15761-1-AP, 1:1000
VPS4A (A-11) Santa Cruz Biotechnology sc-393428, 1:250
MYC (Polyclonal) Thermo Scientific 10828-1-AP, 1:1000
MYC (D84C12) rabbit Cell Signaling Technology 5605S, 1:1000
MYC (C33) mouse Santa Cruz Biotechnology sc-42, 1:500
BRD4 rabbit Bethyl Laboratories A301-985A50, 1:1000
BRD4 (A-7) mouse Santa Cruz Biotechnology sc-518021, 1:500
Cleaved Notch1 (Val1744) (D3B8) Cell Signaling Technology 4147S, 1:1000
Pol II (8WG16) Santa Cruz Biotechnology sc-56767, 1:1000
MED1 Abcam ab64965, 1:1000
P300 (F-4) Santa Cruz Biotechnology sc-48343, 1:500
NR3C1 (D8H2) Cell Signaling Technology 3660T, 1:1000
β-Actin (D6A8) Cell Signaling Technology 8457S, 1:2000
Lamin B1 (D4Q4Z) Cell Signaling Technology 12586S, 1:1000
Vinculin Cell Signaling Technology 4650S, 1:1000
Tubulin Cell Signaling Technology 2144S, 1:1000
FLAG-Tag (M2) Mouse Sigma-Aldrich F1804, 1:2000
FLAG-Tag (D6W5B) Rabbit Cell Signaling Technology 14793S, 1:1000
HA-Tag (C29F4) Rabbit Cell Signaling Technology 3724S, 1:2000
HA-Tag (6E2) Mouse Cell Signaling Technology 2367S, 1:1000
Anti-mouse IgG, HRP linked Antibody Cell Signaling Technology 7076S, 1:5000
Anti-rabbit IgG, HRP linked Antibody Cell Signaling Technology 7074S, 1:5000
RNA pol II Active Motif 39097, 4ug
Histone H3K27ac Active Motif 39085, 4ug
BRD4 (AbFlex) Active Motif 91301, 4ug

	P300 Active Motif 61401, 4ug<br>HA-Tag Abcam ab9110, 4ug<br>Rabbit IgG Cell Signaling Technology 2729S, 4ug<br>Human NGFR APC (ME20.4) BioLegend 345108, 1:100<br>Annexin V APC BioLegend 640941, 1:100<br>Mouse CD45.1 BV421 (A20) BioLegend 110732, 1:100<br>Mouse CD45.2 APC/Fire750 (104) BioLegend 109852, 1:100<br>Mouse CD4 PE/Cy7 (GK1.5) BioLegend 100422, 1:100<br>Mouse CD8 BV786 (53-6.7) BioLegend 100750, 1:100<br>Mouse CD34 AF700 (RAM34) BD Biosciences 560518, 1:100<br>Mouse CD19 BV605 (6D5) BioLegend 115540, 1:100<br>Mouse CD3 AF700 (17A2) BioLegend 100216, 1:100
Validation	All the antibodies used in this study were commercial antibodies. Their validation can be found on the manufacturer's website using the provided catalog number. MYC and CHMP5 antibodies were validated by testing on cells depleted of MYC and CHMP5.

Eukaryotic cell lines

Policy information about cell lines and Sex and Gender in Research

Cell line source(s)	Plat-E (female) from Cell Biolabs (Cat# RV-101), HSB-2 (male) from Sigma Aldrich (Cat# 85112801), CUTLL1 (male) from Adolfo Ferrando at Columbia University. HEK293T (female, Cat# CRL-3216), Jurkat (male, Cat# TIB-152), Loucy (female, Cat# CRL-2629), MOLT-3 (male, Cat# CRL-1552), MOLT-4 (male, Cat# CRL-1582), CCRF-CEM (female, Cat #CCL-119), and SUP-T1 (male, Cat #CRL-1942) were purchased from ATCC; KOPT-K1 (male) and DND-41 (male) from Warren Pear at University of Pennsylvania.
Authentication	Cell lines obtained from commercial sources were authenticated by the vendor through STR profiling. CUTLL1 cells were authenticated by flow cytometry phenotyping. Other cells were authenticated by morphology and western blot.
Mycoplasma contamination	All commonly used cell lines were confirmed negative for mycoplasma over the course of these studies.
Commonly misidentified lines<br>(See ICLAC register)	We did not use any commonly misidentified lines

Animals and other research organisms

Policy information about studies involving animals; ARRIVE guidelines recommended for reporting animal research, and Sex and Gender in Research

Laboratory animals	Six to twelve-week-old male or female B6.SJL-Ptprca Pepcb/BoyJ (B6.SJL), NOD.Cg-Prkdcscid Il2rgtm1Wjl/SzJ (NSG), B6;129-Myctm1Slek/J (MYC-GFP), and B6.Cg-Tg(Cd4-cre)1Cwi/BfluJ (Cd4-Cre) mice were purchased from Jackson Laboratory. Chmp5fl/fl mice (with loxP-flanked exons 3-7 of Chmp5) have been previously described. All mice were maintained in specific-pathogen-free facilities at Case Western Reserve University (Cleveland, OH) or at the NCI campus in Frederick, Maryland under Institutional Animal Care and Use Committee approved protocols.
Wild animals	No wild animals were used in this study
Reporting on sex	This study does not make any conclusions reporting on sex. Both sexes of mice were used as recipients and we observed no differences between sexes.
Field-collected samples	No field-collected samples were used in this study
Ethics oversight	All experimental procedures were approved by the Institutional Animal Care and Use Committee at Case Western Reserve University and the National Institutes of Health.

Note that full information on the approval of the study protocol must also be provided in the manuscript.

Plants

Seed stocks

Report on the source of all seed stocks or other plant material used. If applicable, state the seed stock centre and catalogue number. If plant specimens were collected from the field, describe the collection location, date and sampling procedures.

Novel plant genotypes

Describe the methods by which all novel plant genotypes were produced. This includes those generated by transgenic approaches, gene editing, chemical/radiation-based mutagenesis and hybridization. For transgenic lines, describe the transformation method, the number of independent lines analyzed and the generation upon which experiments were performed. For gene-edited lines, describe the editor used, the endogenous sequence targeted for editing, the targeting guide RNA sequence (if applicable) and how the editor was applied.

Authentication

Describe any authentication procedures for each seed stock used or novel genotype generated. Describe any experiments used to assess the effect of a mutation and, where applicable, how potential secondary effects (e.g. second site T-DNA insertions, mosiacism, off-target gene editing) were examined.

ChIP-seq

Data deposition

☒ Confirm that both raw and final processed data have been deposited in a public database such as GEO.

☒ Confirm that you have deposited or provided access to graph files (e.g. BED files) for the called peaks.

Data access links
May remain private before publication.

https://www.ncbi.nlm.nih.gov/geo/query/acc.cgi?acc=GSE244197

Files in database submission

GSM7808516 CUTLL1 BRD4 CT
GSM7808517 CUTLL1 BRD4 KD
GSM7808518 CUTLL1 Pol II CT
GSM7808519 CUTLL1 Pol II KD
GSM7808520 CUTLL1 H3K27ac CT
GSM7808521 CUTLL1 H3K27ac KD
GSM7808522 CUTLL1 CT 2% input
GSM7808523 CUTL1 KD 2% input

Genome browser session
(e.g. UCSC)

https://genome.ucsc.edu/s/sratnayake/ChIPseq_CT_KD

Methodology

Replicates

2

Sequencing depth

ChIP samples were sequenced in paired-end mode with sequencing length of 150bp.

Antibodies

RNA pol II (Clone 4H8) Active Motif 39097
Histone H3K27ac (Clone MABI 0309) Active Motif 39085
BRD4 (AbFlex) Active Motif 91301

Peak calling parameters

Sequencing reads from samples were aligned to the reference genome UCSC hg38 with Bowtie2 (v2.4.5) with –local setting and unique reads were kept by filtering out unmapped, duplicated and multimapped reads with sambamba 0.8.2 custom filters -F "[XS] == null and not unmapped and not duplicate". MACS2 (v2.7.1)with default parameters was used for peak calling over the corresponding background. MACS2 peaks were annotated with ChIPseeker (v1.34.1) with TSS defined from -1kb to +1kb.

Data quality

Describe the methods used to ensure data quality in full detail, including how many peaks are at FDR 5% and above 5-fold enrichment.

Software

Bowtie2 (v2.4.5) for ChIP sequencing alignment
MACS2 (v2.7.1) for peak calling of ChIP-seq data
ChIPseeker (v1.34.1) for peak annotation
ngsplot for ChIP-seq metaplots
UCSC genome browser for ChIP track generation
ROSE software for enhancer mapping
PIC software for traveling ratio calculation

Flow Cytometry

Plots

Confirm that:

- [x] The axis labels state the marker and fluorochrome used (e.g. CD4-FITC).
- [x] The axis scales are clearly visible. Include numbers along axes only for bottom left plot of group (a 'group' is an analysis of identical markers).
- [x] All plots are contour plots with outliers or pseudocolor plots.
- [x] A numerical value for number of cells or percentage (with statistics) is provided.

Methodology

Sample preparation	Bone marrow, thymus and spleen from mice were harvested and processed by mechanical dissociation to obtain single cells. Blood was collected either from the tail vein or medial saphenous vein into K2 EDTA coated collection tubes (BD Biosciences #365974). Splenocytes, blood, and bone marrow cells were treated with ACK lysis buffer (Gibco #A1049201) to lyse red blood cells. Single-cell suspensions were filtered through 40-µm strainers and resuspended in complete RPMI. CUTLL1 cells were harvested from culture.
Instrument	BD LSR Fortessa and BD LSR-II
Software	BD FACSDiva for collection and FlowJo for data analysis
Cell population abundance	Murine T-ALL cells (CD45.2+ NGFR+) from ICN1 chimera mice were between 4-80% of total live lymphocytes. For sorting, sorted cells were checked for post-sort purity above 90%.
Gating strategy	Cells were first gated on lymphocytes (FSC-A between 30-200K, SSC-A between 0-200K) then live cells were gated on DAPI negative population. Single cells were gated on FSC-W by FSC-H and SSC-W by SSC-H. CD45.2+ and NGFR+ cells were gated on this population (Supplementary Figure 7d) CUTLL1 cells for metabolic studies used this same gating strategies.

- [x] Tick this box to confirm that a figure exemplifying the gating strategy is provided in the Supplementary Information.
